# Impacts of obstacle-crossing during walking on postural control strategies in individuals with functional ankle instability

**DOI:** 10.3389/fbioe.2025.1650015

**Published:** 2025-08-15

**Authors:** Ke Ma, Wenlong Zhou, Xiangwei Shi, Guodong Wang, Xiaokun Mao, Lingyu Kong, Qiuxia Zhang

**Affiliations:** School of Physical Education and Sports, Soochow University, Suzhou, China

**Keywords:** FAI, obstacle height, biomechanics, musculoskeletal injury, margin of stability, postural control strategies

## Abstract

**Background:**

Lateral ankle sprains often progress to functional ankle instability (FAI). Obstacle-crossing could pose greater challenges for individuals with FAI due to significant impairments in ankle kinesthesia and joint position sense. While existing studies have focused on level-ground gait characteristics in FAI, the postural control strategies underlying obstacle-crossing remain unclear, and the impact of obstacle height on these strategies has not been investigated.

**Purpose:**

This study is aimed at analyzing the postural control strategies of individuals with FAI during obstacle-crossing at different heights.

**Methods:**

Twenty-three male participants [unilateral FAI group (n = 11) and matched controls (n = 12)] were recruited. FAI was identified using the Cumberland Ankle Instability Tool (CAIT score <24). Obstacle heights were set at 0%, 10%, and 20% of individuals’ leg length (LL). Participants completed crossing tasks in randomized order. The individuals with FAI use their FAI-affected limb as the swing leg and controls use the matched limb.

**Results:**

Compared to the control group, the FAI group exhibited smaller hip flexion angles (*P* = 0.008), greater trunk lateral flexion (*P* = 0.033), and reduced medio-lateral margin of stability (ML_MoS) at landing (*P* = 0.046). As obstacle height increased, the FAI group showed significant differences in ML_MoS at landing (*P* < 0.001), with notably lower ML_MoS when the obstacle height was set at 20% LL compared to controls (*P* = 0.001).

**Conclusion:**

Compared to healthy individuals, those with FAI adapt movement patterns through proximal compensation strategies, characterized by compensatory trunk lateral flexion. Increased obstacle height exacerbates instability during landing, particularly at higher heights, where individuals with FAI demonstrate significantly diminished lateral stability. These findings emphasize the critical influence of FAI on balance control and adaptive postural control strategies during obstacle-crossing.

## 1 Introduction

Lateral ankle sprains, one of the most common musculoskeletal injuries, have a prevalence of 11.88% in the general population and account for approximately 20% of all sports injuries (Doherty et al., 2013; Wagemans et al., 2022). There is consensus that the initial injuries that are not treated properly can lead to chronic musculoskeletal problems. In most cases, individuals with functional ankle instability (FAI) experience difficulty making a full recovery within 3 years (van Rijn et al., 2008). Furthermore, the risk of recurrent injuries is significantly increased (Hung, 2015; Docherty et al., 2006), which ultimately evolves into FAI (Beynnon et al., 2001). FAI-induced damage to mechanoreceptors in the lateral ankle ligaments (e.g., the anterior talofibular ligament) causes abnormal proprioceptive input (Arnold et al., 2011), which has been demonstrated to trigger progressive hyperalgesia of ankle positional, kinesthetic, and force sensations (Liu et al., 2024). As the disease progresses, this neurosensory dysfunction evolves into reduced muscle strength and neuromuscular control (Cruz-Montecinos et al., 2025; Akbari and Yousefi, 2023). Previous studies have revealed that individuals with FAI may adopt compensatory strategies during walking, such as increased hip and knee joint mobility, to maintain dynamic balance (Akbari and Yousefi, 2023). While this abnormal gait pattern partially compensates for ankle function deficits, it may elevate injury risks under certain circumstances (Son et al., 2019). Individuals with FAI have been found to have a higher incidence of falls in the past 12 months and a greater prevalence of fall-related injuries and hospitalizations compared to those without FAI (Al Mahrouqi et al., 2023).

Improper crossing of obstacles during walking is a critical risk factor for falls (Al Mahrouqi et al., 2023). Compared to level walking, obstacle-crossing imposes higher demands on neuromuscular control, requiring not only enhanced activation of specific muscles (e.g., knee flexors) and refined allocation of cognitive-sensory resources but also significant increases in prefrontal cortex activity, reflecting heightened neuromuscular and central regulatory demands (Patla et al., 1996; Patla et al., 1991; MacLellan and McFadyen, 2013; Chen et al., 2017). Consequently, intact proprioception is essential for successful obstacle-crossing (Billington et al., 2013). Ligament injuries in individuals with FAI damage proprioceptive nerve endings and trigger reorganization within the central nervous system’s motor cortex. This impairs multiple facets of proprioception, including position sense, movement sense, force sense, and vibration sensation (Peng et al., 2024; Ward et al., 2015). Research indicates a significant negative correlation between CAIT scores and inversion proprioception (Peng et al., 2024; Xue et al., 2021). Crucially, the concurrent integration deficits in multiple proprioceptive domains compromise precise foot clearance height regulation and landing impact attenuation, ultimately elevating injury risk.

Research further indicates that compared to healthy individuals, those with FAI display reduced ankle dorsiflexion, increased inversion, and elevated knee internal rotation moments during walking and jogging (Lee et al., 2022). Additionally, individuals with FAI frequently exhibit neuromuscular control deficits, such as prolonged reaction times in the peroneus longus muscle (Méndez-Rebolledo et al., 2015). They also demonstrate impaired control of ankle muscle force output, suggesting a potential link between FAI and diminished ability to perceive force generation (Yen et al., 2019). Furthermore, weakness in muscle groups surrounding other lower limb joints may compromise ankle stability in individuals with FAI (Yeum et al., 2024). Thus, analyzing lower limb joint kinematics during obstacle-crossing in FAI populations could help identify potential postural control deficits. Although existing evidence implies that individuals with FAI may adopt abnormal gait patterns during crossing obstacles, no studies have yet investigated the postural control strategies of obstacle-crossing in this population, leaving the impact of obstacle-crossing on individuals with FAI poorly understood.

As obstacle height increases, the complexity of obstacle-crossing rises significantly (Huang et al. 2008). During dynamic locomotion, it is necessary to appropriately maintain the extrapolated center of mass (XCoM) within the base of support (BoS) to enhance stability (Hof et al., 2005). The margin of stability (MoS) serves as an effective metric for analyzing the dynamic relationship between the XCoM and the BoS (AminiAghdam et al., 2019). As previously discussed, given that individuals with FAI exhibit proprioceptive deficits, elevated obstacle heights likely impose greater demands on their neuromuscular control capacity. These individuals must precisely regulate gait parameters (e.g., step length, walking speed, and body posture) to meet the challenge, creating dual pressure on their already compromised motor control abilities (Wang et al., 2007; Galna et al., 2010; Rosker et al., 2024). Additionally, to compensate for distal joint control deficits, individuals with FAI often adopt compensatory strategies by increasing proximal joint (e.g., hip and trunk) movements to maintain dynamic balance (Huang et al., 2020). However, current research has yet to systematically explore the mechanisms by which obstacles of varying heights affect individuals with FAI, leaving a knowledge gap regarding the postural control strategies of their obstacle-crossing strategies across different obstacle heights.

Therefore, to prevent fall-related injuries that may disrupt daily activities and incur economic burdens, it is essential to investigate the postural control strategies of obstacle-crossing in individuals with FAI. This study aims to examine the postural control strategies of walking while crossing obstacles of different heights in individuals with FAI, specifically analyzing the impact of obstacle heights relative to leg length (LL). To the best of our knowledge, this study may be the first to systematically quantify how progressive increases in obstacle height (0%, 10%, 20% LL) exacerbate postural control deficits in individuals with FAI during obstacle-crossing, thereby addressing the critical research gap regarding the dose-response relationship between obstacle height and dynamic stability. The findings will provide theoretical insights to guide the development of healthy behavioural awareness, fall prevention strategies, and injury avoidance in daily activities for individuals with FAI. The specific objectives of this study are:

1. To clarify the postural control strategies of obstacle-crossing in individuals with FAI.
2. To explore how obstacle height influences obstacle-crossing strategies in individuals with FAI compared to healthy controls.

To address the above objectives, this study proposes the following two hypotheses based on existing literature and prior research findings:

Hypothesis 1 The postural control strategies during obstacle-crossing tasks may differ between individuals with FAI and healthy controls. Individuals with FAI may be unable to effectively utilize distal joints and may rely compensatorily on proximal joints to maintain posture during obstacle-crossing.

Hypothesis 2 Increased obstacle height will induce changes in postural control strategies, and individuals with Individuals with FAI will face greater postural control challenges compared to healthy individuals as obstacle height rises, which may exacerbate stability deficits in individuals with FAI during landing.

## 2 Methods

### 2.1 Sample size calculation

The required sample size was calculated using G*Power (Version 3.1.9, Heinrich Heine University Disseldorf, Germany). We estimated the sample size for both the group main effect and the group-by-condition interaction effect. Based on our research group’s previous study comparing individuals with FAI and healthy controls (Wang et al., 2024a; Wang et al., 2024b), the *η* ^*2*^ values for the group main effect ranged from 0.224 to 0.542, while those for the group-by-condition interaction effect ranged from 0.128 to 0.283. Intermediate *η* ^*2*^ values were selected to determine the effect sizes: 0.383 for the group main effect and 0.206 for the interaction effect. Using a significance level of *α* = 0.05 and a desired statistical power of 1 − *β* = 0.8, the calculation indicated a minimum requirement of 12 participants for the group main effect size and 10 participants for the interaction effect size. Consequently, the more conservative estimate of 12 participants was adopted. To account for a potential 20% rate of invalid samples, the target sample size was increased to 15 participants.

### 2.2 Participants

This study recruited 12 males with unilateral FAI as the experimental group through the Cumberland Ankle Instability Tool (CAIT) (Hiller et al., 2006) and anterior drawer test, along with 12 healthy males matched for age, height, weight, and other demographic criteria as the control group. Due to equipment malfunction, data from one participant were excluded, resulting in a final sample of 23 participants. The baseline characteristics of the two groups are summarized in Table 1. All participants provided written informed consent, and the study was approved by the Ethics Committee of Soochow University (Ethics Approval No: SUDA20250327H01).

**TABLE 1:** Characteristics of participants (mean ± SD).

Variables	FAI (n = 11)	Control (n = 12)	*P*-value
Age (year)	24.1 ± 0.8	24.3 ± 0.9	0.598
Height (m)	1.76 ± 0.05	1.73 ± 0.05	0.177
Weight (kg)	73.0 ± 6.8	70.0 ± 6.3	0.277
BMI (kg/m^2^)	23.7 ± 2.4	23.4 ± 2.2	0.643
CAIT (score)	17.27 ± 4.15	27.75 ± 1.60	<0.001*
LL (m)	0.88 ± 0.05	0.87 ± 0.05	0.795

Note: *: *P* < 0.05; Independent t-tests confirmed no group differences in age, height, weight, or BMI (all *P* > 0.05), except CAIT, scores (*P* < 0.001).

Abbreviations: FAI, Functional Ankle Instability; CAIT, Cumberland ankle instability tool; LL, leg length.

Inclusion and exclusion criteria were based on previous literature. Inclusion criteria for the FAI group included at least one ankle sprain in the past year with self-reported instability (Ardakani et al., 2019); A CAIT score below 24 (Donahue et al., 2011); No history of severe lower limb injuries (e.g., fractures or major orthopedic trauma), excluding ankle sprains (Wu et al., 2022); and Unilateral FAI. Exclusion criteria for both groups were: History of bilateral ankle sprains (Wang et al., 2022); Acute lower limb pathologies; Prior lower limb surgeries (Kweon et al., 2022); Balance dysfunction (Donahue et al., 2011); Congenital deformities of the feet, ankles, knees, pelvis, or spine; Positive talar tilt test and/or anterior drawer test results in either ankle.

### 2.3 Experimental equipment

**FIGURE 1:**
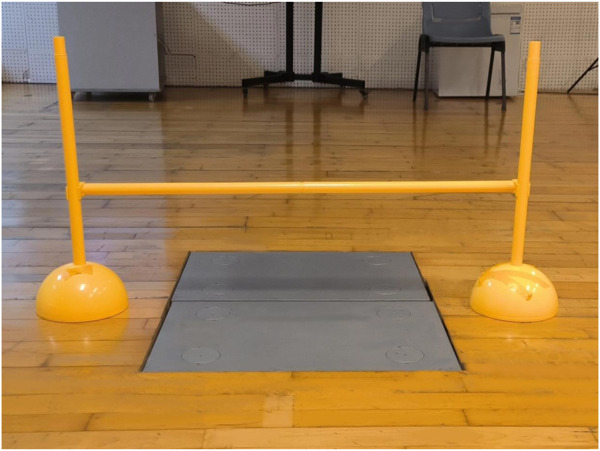
Adjustable-height assembled poles used as the obstacle apparatus in the experiment.

This study utilized an 8-camera infrared motion capture system (Vicon, United Kingdom) synchronized with two three-dimensional force plates (9281, Kistler, Switzerland) to collect kinematic and kinetic data during obstacle-crossing tasks. The Vicon system recorded body movement trajectories at a sampling frequency of 100 Hz, while the force plates captured ground reaction force parameters at 1,000 Hz. An adjustable-height obstacle frame assembly (AOTII, China) was employed to create obstacle heights at 0%, 10%, and 20% of each participant’s LL (Figure 1).

### 2.4 Experimental protocol

#### 2.4.1 Pre-test preparation

**FIGURE 2:**
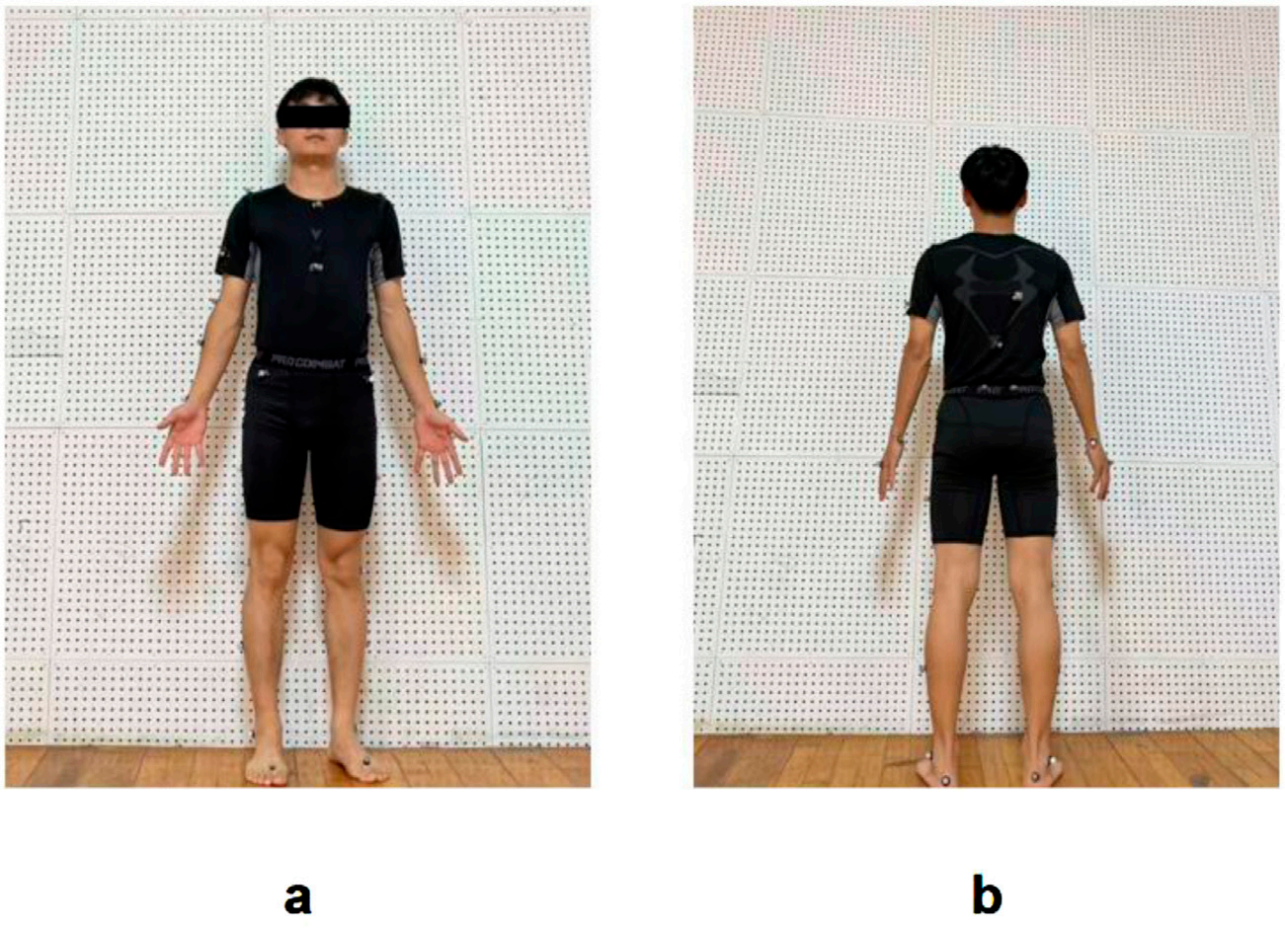
Marker placement configuration for the Plug-in Gait full-body model: **(a)** Anterior view; **(b)** Posterior view.

First, participants’ anthropometric measurements (height, weight, and LL) were recorded. Marker placement (39 markers in total) adhered to the Plug-in Gait full-body model (Vicon, Oxford, United Kingdom), with reflective markers positioned at key anatomical landmarks including the head, trunk (C7/T10/sternum/clavicle), pelvis (anterior/posterior superior iliac spines), upper extremities (shoulders/elbows/wrists), and lower extremities (thighs/knees/shanks/ankles/heels/toes). Marker placements are detailed in Figure 2. To minimize variability, all markers were positioned by the same researcher, who also collected morphological data prior to testing.

#### 2.4.2 Obstacle-crossing task

Before dynamic data collection, static calibration trials were performed following standardized procedures. Participants then completed obstacle-crossing trials under three height conditions: 0%, 10%, and 20% of their LL. The order of obstacle heights was randomized. Each condition was repeated multiple times (with a minimum 30-s rest between trials) until three valid trials per condition were obtained (Chardon et al., 2024; Lee et al., 2022). A valid trial was defined as one where: (1) the participant successfully crossed the obstacle during the task, (2) motion capture data from all markers was successfully collected, enabling the generation of a complete biomechanical model, (3) at the moment of swing leg touchdown, the foot remained completely within the boundaries of the force plate, and (4) no abnormal signals were contained in the data during preprocessing. Based on this procedure, we prevented the inclusion of potential outliers, as any aberrant data points were excluded during our experimental process.

**FIGURE 3:**
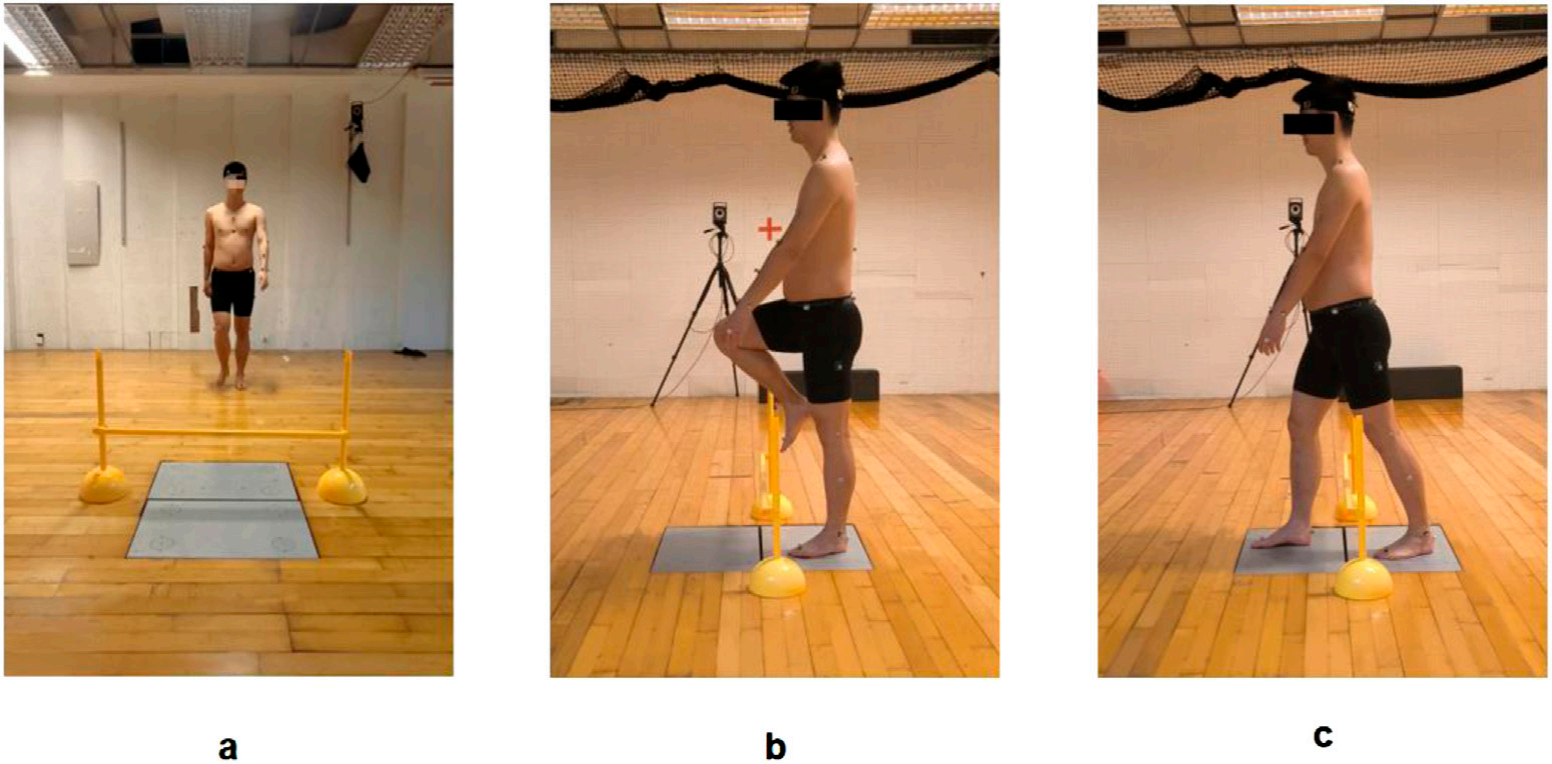
Obstacle-crossing test during walking: **(a)** Starting position; **(b)** Mid-crossing phase; **(c)** Completion phase.

The task protocol was as follows: Participants stood 3 m in front of the Kistler force plates in a natural upright posture, gazing forward. Upon the “start” command, they walked forward at a self-selected comfortable speed, crossed the obstacle, and continued walking 5 m beyond the force plates. During the crossing, the support leg (FAI-affected limb in the experimental group or matched limb in controls) contacted the proximal force plate, while the swing leg landed on the distal force plate (Figure 3). Three successful trials were collected for each obstacle height, with the FAI-affected limb (or matched limb in controls) consistently used as the swing leg during the crossing.

### 2.5 Data analysis

The full-body skeletal model was defined in Vicon Nexus software, and the Plug-in Gait Full Body model (15 rigid segments: head, trunk, pelvis, bilateral thighs, shanks, feet, upper arms, forearms, and hands) was reconstructed. Motion capture data and ground reaction forces were processed with a Butterworth low-pass filter (cutoff frequencies: 6 Hz for kinematics and 50 Hz for kinetics) and analyzed using custom MATLAB scripts to extract relevant parameters (Zhang and Zhang, 2023).

#### 2.5.1 Spatiotemporal gait parameters

The obstacle-crossing task was defined as the period from the moment the swing leg leaves the ground to when it makes contact with the ground again after crossing the obstacle. This period was further divided into three phases: (1) Preparation phase, defined as the interval from when the swing leg toe lifts more than 3 cm above the ground (while the supporting leg remains in contact with the proximal force plate) to when the swing leg reaches a position within ±1 cm in front of the obstacle; (2) Execution phase, spanning from the instant the swing leg reaches ±1 cm anterior to the obstacle to the point the heel passes at least 1 cm beyond the obstacle; (3) Completion phase, which is from the heel extending 1 cm beyond the obstacle until the swing leg contacts the distal force plate (ground reaction force >10 N).

For each phase and the entire obstacle-crossing period, both phase-specific and total crossing velocities were calculated, with the overall velocity determined as the obstacle length divided by total crossing time. The proportion of time spent in each phase relative to the entire swing phase was also analyzed. Additionally, spatial parameters were assessed, including step width and length (measured as the mediolateral and anteroposterior distances between heel markers at initial contact), vertical clearance (the vertical distance between the swing foot’s toe and heel markers and the obstacle during the execution phase), and horizontal distance (the anteroposterior distance between the support leg’s toe marker and the swing leg’s heel marker relative to the obstacle at task completion).

#### 2.5.2 Joint kinematic parameters

Joint angles were calculated in local anatomical coordinate systems according to the Plug-in Gait standards. The swing phase of the swinging leg was rescaled to a normalized 0%–100% time base, and the mean joint angle across this normalized swing phase was extracted for group comparisons. For the ankle, dorsiflexion (sagittal plane) and inversion (frontal plane) were defined as positive, while plantarflexion (sagittal plane) and eversion (frontal plane) were defined as negative values. For the hip, extension (sagittal) and adduction (frontal) were negative, while flexion (sagittal) and abduction (frontal) were positive. For the knee, extension was negative, and flexion was positive in the sagittal plane. For the trunk, flexion was positive and extension negative in the sagittal plane, while medio flexion was negative and lateral flexion positive in the frontal plane.

#### 2.5.3 Dynamic stability metrics

**FIGURE 4:**
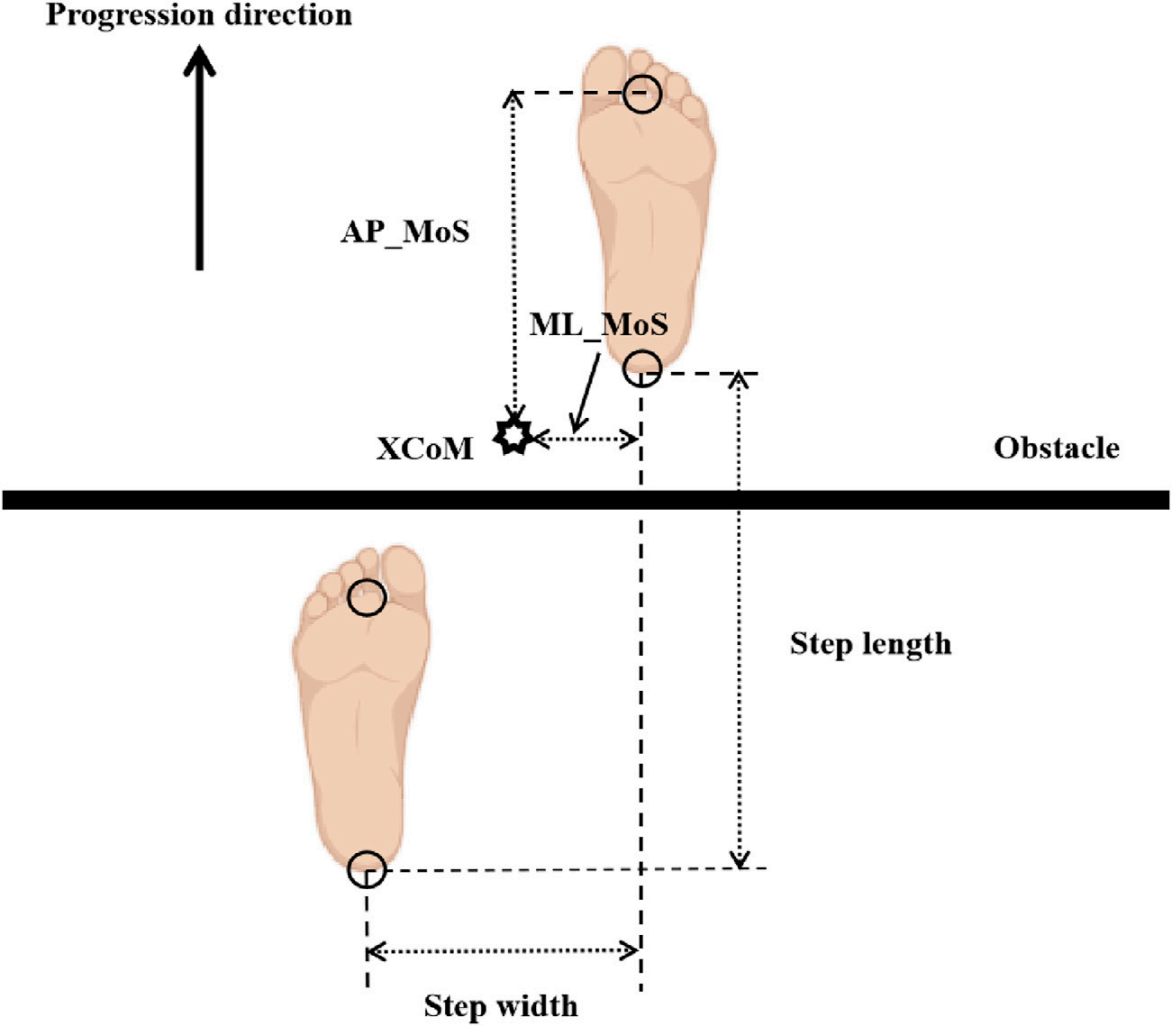
Definitions of spatial gait parameters and margin of stability (MoS). Foot markers indicate the position of each foot at the instant of swing limb heel contact. The arrow denotes the direction of walking progression. Step length = anterior-posterior (AP) distance between the two heel markers; step width = medial-lateral (ML) distance between the two heel markers. AP_MoS = AP distance between the XCoM and the toe marker of the support leg (Preparation phase/Execution phase) or the toe marker of the swing leg (Completion phase); ML_MoS = lateral distance between the XCoM and the toe marker of the support leg (Preparation phase/Execution phase) or the heel marker of the swing leg (Completion phase).

To quantitatively reflect the participants’ ability to maintain dynamic balance upon landing after obstacle crossing, the MoS at the moment when the swing leg lands after crossing the obstacle was used (AminiAghdam et al., 2019). By Equation 1 the MoS represents the shortest distance from the horizontal projection of the body’s XCoM to the nearest boundary of the BoS. The calculation of XCoM involves determining the dynamic position of the center of mass (CoM). And we computed the Medio-lateral Margin of Stability (ML_MoS) and the Anterior-posterior Margin of Stability (AP_MoS) are defined, see Equations 2, 3. These spatial and postural stability variables are illustrated in Figure 4. Following prior literature (Qu et al., 2021), XCoM, ML_MoS and AP_MoS were calculated as:

XCoM=CoM+VELCoM×lg (1)

ML_MoS=BoS_ML ‐ XCoM_ML (2)

AP_MoS=BoS_AP ‐ XCoM_AP (3)

where: CoM represents the whole-body CoM displacement and was obtained from the Plug in Gait model output, VEL_CoM_ is the velocity of the CoM, *l* is the vertical distance from the CoM to the ground, and *g* = 9.81 m/s^2^ is the acceleration due to gravity. When calculating the ML_MoS and AP_MoS, the edge of the BoS can be judged by the marker at the heel. Positive MoS values indicate CoM position within the BoS, while negative values indicate instability (CoM outside BoS). Positive MoS values indicate CoM position within the BoS, while negative values indicate instability (CoM outside BoS) (Hof et al., 2005).

#### 2.5.4 Statistical analysis

Data analysis was performed using SPSS 27.0. Continuous variables are presented as mean ± standard deviation (SD). A mixed ANOVA was conducted to evaluate the main effects of obstacle height (within-subject factor: 0% LL, 10% LL, 20% LL), the main effects of group (between-subject factor: FAI vs. control), and interaction effects between group and obstacle height. If a significant interaction was detected, a simple effects analysis was performed to compare differences between groups at each obstacle height. Statistical significance was set at α = 0.05. *Post hoc* comparisons were adjusted using the Bonferroni correction.

## 3 Results

Table 2 presents the spatiotemporal parameters. Significant main effects of obstacle height were observed for step length (*F* = 5.156, *P* = 0.022, *η* ^*2*^ = 0.197), swing total speed (*F* = 130.267, *P* < 0.001, *η* ^*2*^ = 0.861), swing pre speed (*F* = 95.016, *P* < 0.001, *η* ^*2*^ = 0.819), swing obstacle speed (*F* = 158.379, *P* < 0.001, *η* ^*2*^ = 0.883), swing post speed (*F* = 158.508, *P* < 0.001, *η* ^*2*^ = 0.883), the proportion of the preparation phase (*F* = 47.061, *P* < 0.001, *η* ^*2*^ = 0.691), the proportion of the execution phase (*F* = 20.946, *P* < 0.001, *η* ^*2*^ = 0.499), and the proportion of the completion phase (*F* = 44.987, *P* < 0.001, *η* ^*2*^ = 0.682). Post hoc comparisons revealed the following trends as obstacle height increased: From 0% to 10% and 20% LL, swing total speed decreased by 0.215 m/s (*P* < 0.001) and 0.264 m/s (*P* < 0.001). Swing pre-speed decreased by 0.175 m/s (*P* < 0.001) and 0.218 m/s (*P* < 0.001). Swing obstacle speed decreased by 0.259 m/s (*P* < 0.001) and 0.314 m/s (*P* < 0.001). Swing post speed decreased by 0.250 m/s (*P* < 0.001) and 0.301 m/s (*P* < 0.001). The proportion of the preparation phase decreased by 7.794% (*P* < 0.001) and 9.760% (*P* < 0.001). The proportion of the execution phase decreased by 2.863% (*P* < 0.001) and 3.524% (*P* < 0.001). The proportion of the completion phase increased by 10.657% (*P* < 0.001) and 13.283% (*P* < 0.001). From 10% to 20% LL, Swing total speed decreased by 0.049 m/s (*P* < 0.001). Swing pre speed decreased by 0.042 m/s (*P* = 0.002). Swing obstacle speed decreased by 0.054 m/s (*P* < 0.001). Swing post speed decreased by 0.051 m/s (*P* < 0.001). The proportion of the preparation phase decreased by 1.965% (*P* = 0.011). The proportion of the completion phase increased by 2.626% (*P* < 0.001). No significance was found in the other metrics. No significant between-group differences or interaction effects were observed in the obstacle distance-related parameters during obstacle-crossing.

**TABLE 2:** Comparison of step length, step width, velocity, and phase proportions between the FAI group and control group during walking while crossing obstacles of different heights (mean ± SD).

Variables	Height	Group		*P*-value		
		FAI	Control	Group	Height	Group × height
Step width (m)	0%	0.12 ± 0.03	0.13 ± 0.05	0.148	0.061	0.240
	10%	0.12 ± 0.03	0.15 ± 0.07			
	20%	0.10 ± 0.04	0.14 ± 0.06			
Step length (m)	0%	0.43 ± 0.04	0.44 ± 0.04	0.257	0.022*	0.862
	10%	0.45 ± 0.03	0.47 ± 0.03			
	20%	0.45 ± 0.04	0.47 ± 0.02			
Swing total speed (m/s)	0%	0.95 ± 0.14	0.99 ± 0.14	0.787	<0.001*^abc^	0.279
	10%	0.76 ± 0.13	0.75 ± 0.14			
	20%	0.70 ± 0.11	0.71 ± 0.14			
Swing pre speed (m/s)	0%	0.96 ± 0.13	0.99 ± 0.14	0.875	<0.001*^abc^	0.322
	10%	0.80 ± 0.12	0.79 ± 0.14			
	20%	0.76 ± 0.11	0.76 ± 0.13			
Swing obstacle speed (m/s)	0%	0.93 ± 0.14	0.98 ± 0.13	0.731	<0.001*^abc^	0.266
	10%	0.70 ± 0.14	0.69 ± 0.15			
	20%	0.63 ± 0.12	0.64 ± 0.15			
Swing post speed (m/s)	0%	0.93 ± 0.14	0.99 ± 0.13	0.718	<0.001*^abc^	0.138
	10%	0.72 ± 0.14	0.70 ± 0.15			
	20%	0.66 ± 0.11	0.66 ± 0.14			
Proportion of the preparation phase (%)	0%	59.04 ± 5.03	59.06 ± 5.99	0.229	<0.001*^abc^	0.336
	10%	50.05 ± 4.16	52.46 ± 4.20			
	20%	47.84 ± 3.78	50.73 ± 3.52			
Proportion of the execution phase (%)	0%	17.49 ± 3.04	15.56 ± 2.38	0.177	<0.001*^ab^	0.186
	10%	13.52 ± 1.61	13.81 ± 1.55			
	20%	13.44 ± 2.03	12.57 ± 1.90			
Proportion of the completion phase (%)	0%	23.47 ± 7.31	25.38 ± 7.46	0.585	<0.001*^abc^	0.253
	10%	36.43 ± 4.77	33.73 ± 4.68			
	20%	38.72 ± 4.97	36.70 ± 4.48			

Note: *: *P* < 0.05; ^a^: Significant difference when obstacle height increased from 0% to 10%; ^b^: Significant difference when obstacle height increased from 0% to 20%; ^c^: Significant difference when obstacle height increased from 10% to 20%.

Abbreviation: FAI, Functional Ankle Instability.

Table 3 displays foot-to-obstacle distance metrics during obstacle-crossing. Significant main effects of obstacle height were observed for vertical clearance of right toe (*F* = 233.764, *P* < 0.001, *η* ^*2*^ = 0.918), vertical clearance of right heel (*F* = 178.532, *P* < 0.001, *η* ^*2*^ = 0.895), vertical clearance of left toe (*F* = 220.062, *P* < 0.001, *η* ^*2*^ = 0.913), vertical clearance of left heel (*F* = 224.172, *P* < 0.001, *η* ^*2*^ = 0.914), horizontal distance of right heel (*F* = 5.760, *P* = 0.011, *η* ^*2*^ = 0.215), horizontal distance of left toe (*F* = 3.667, *P* = 0.049, *η* ^*2*^ = 0.149). Post hoc comparisons revealed the following trends as obstacle height increased: From 0% to 10% and 20% LL, Vertical clearance of right toe increased by 0.204 m (*P* < 0.001) and 0.165 m (*P* < 0.001). Vertical clearance of right heel increased by 0.192 m (*P* < 0.001) and 0.155 m (*P* < 0.001). Vertical clearance of left toe increased by 0.236 m (*P* < 0.001) and 0.200 m (*P* < 0.001). Vertical clearance of left heel increased by 0.246 m (*P* < 0.001) and 0.208 m (*P* < 0.001). Horizontal distance of right heel increased by 0.023 m (*P* = 0.036) and 0.021 m (*P* = 0.067). From 10% to 20% LL, Vertical clearance of right toe increased by 0.039 m (*P* = 0.001). Vertical clearance of right heel decreased by 0.037 m (*P* = 0.001). Vertical clearance of left toe decreased by 0.036 m (*P* = 0.001). Vertical clearance of left heel decreased by 0.038 m (*P* = 0.004). No significance was found in the other metrics. And no significant between-group differences or interaction effects were observed in the obstacle distance-related parameters during obstacle-crossing.

**TABLE 3:** Comparison of foot-to-obstacle distances between the FAI group and control group during walking while crossing obstacles of different heights (mean ± SD).

Variables	Height	Group		*P*-value		
		FAI	Control	Group	Height	Group × height
Vertical clearance of right toe (m)	0%	0.07 ± 0.01	0.07 ± 0.01	0.320	<0.001*^abc^	0.769
	10%	0.28 ± 0.06	0.26 ± 0.05			
	20%	0.24 ± 0.04	0.23 ± 0.03			
Vertical clearance of right heel (m)	0%	0.09 ± 0.02	0.09 ± 0.01	0.554	<0.001*^abc^	0.776
	10%	0.29 ± 0.06	0.27 ± 0.06			
	20%	0.25 ± 0.06	0.24 ± 0.05			
Vertical clearance of left toe (m)	0%	0.09 ± 0.01	0.09 ± 0.01	0.708	<0.001*^abc^	0.996
	10%	0.33 ± 0.07	0.32 ± 0.05			
	20%	0.29 ± 0.06	0.29 ± 0.04			
Vertical clearance of left heel (m)	0%	0.26 ± 0.02	0.25 ± 0.02	0.289	<0.001*^abc^	0.885
	10%	0.51 ± 0.07	0.49 ± 0.05			
	20%	0.47 ± 0.04	0.46 ± 0.05			
Horizontal distance of right heel (m)	0%	0.08 ± 0.04	0.10 ± 0.03	0.271	0.011*^a^	0.344
	10%	0.11 ± 0.03	0.11 ± 0.03			
	20%	0.10 ± 0.03	0.12 ± 0.03			
Horizontal distance of left toe (m)	0%	0.19 ± 0.03	0.20 ± 0.03	0.263	0.049*	0.417
	10%	0.17 ± 0.03	0.19 ± 0.02			
	20%	0.18 ± 0.03	0.18 ± 0.03			

Note: *: *P* < 0.05; a: Significant difference when obstacle height increased from 0% to 10%; b: Significant difference when obstacle height increased from 0% to 20%; c: Significant difference when obstacle height increased from 10% to 20%.

Abbreviation: FAI, Functional Ankle Instability.

Table 4 presents the results regarding joint angles. Main effects of obstacle height were found in ankle sagittal plane angle (*F* = 6.781, *P* = 0.010, *η* ^2^ = 0.224), ankle frontal plane angle (*F* = 18.179, *P* < 0.001, *η* ^2^ = 0.464), hip sagittal plane angle (*F* = 660.805, *P* < 0.001, *η* ^2^ = 0.969), hip frontal plane angle (*F* = 10.041, *P* = 0.002, *η* ^2^ = 0.323), knee sagittal plane angle (*F* = 601.895, *P* < 0.001, *η* ^2^ = 0.167), trunk sagittal plane angle (*F* = 14.976, *P* < 0.001, *η* ^2^ = 0.416), and trunk frontal plane angle (*F* = 4.638, *P* = 0.015, *η* ^2^ = 0.181). Post-hoc comparisons revealed that as obstacle height increased from 0% to 10% and 20%, ankle plantar flexion decreased by 2.4° (*P* = 0.025) and 1.8° (*P* = 0.088), ankle inversion increased by 0.9° (*P* < 0.001) and 0.8° (*P* = 0.001), hip flexion increased by 25.1° (*P* < 0.001) and 27.8° (*P* < 0.001), hip adduction increased by 1.9° (*P* = 0.027) and 2.6° (*P* = 0.005), knee flexion increased by 40.1° (*P* < 0.001) and 43.2° (*P* < 0.001), trunk extension decreased by 2.0° (*P* < 0.001) and 2.3° (*P* = 0.002), and trunk lateral flexion increased by 0.1° (*P* = 1.000) and 0.8° (*P* = 0.023). When obstacle height increased from 10% to 20%, hip flexion increased by 2.8° (*P* = 0.002), and knee flexion increased by 3.1° (*P* = 0.021), while no significant differences were found in other parameters. Group differences were observed in hip sagittal plane angle (*F* = 8.642, *P* = 0.008, *η* ^2^ = 0.292) and trunk frontal plane angle (*F* = 5.239, *P* = 0.033, *η* ^2^ = 0.200), with post-hoc analysis showing the FAI group had significantly smaller hip flexion angles (7.3°, *P* = 0.008) and greater trunk lateral flexion angles (1.5°, *P* = 0.033) than the control group. No interaction effects were observed in any joint angle measures.

**TABLE 4:** Comparison of ankle, hip, knee, and trunk joint angles between the FAI group and control group during walking over obstacles of different heights (mean ± SD).

Variables	Height	Group		*P*-value		
		FAI	Control	Group	Height	Group × height
Ankle dorsiflexion (+)/plantar flexion (−) (°)	0%	−11.0 ± 4.7	−12.7 ± 2.5	0.478	<0.010*^a^	0.478
	10%	−9.3 ± 3.9	−9.5 ± 3.9			
	20%	−9.5 ± 4.3	−10.6 ± 3.4			
Ankle inversion(+)/eversion (−) (°)	0%	−0.4 ± 1.7	0.0 ± 0.8	0.715	<0.001*^ab^	0.550
	10%	0.6 ± 2.0	0.8 ± 1.2			
	20%	0.6 ± 2.0	0.8 ± 1.2			
Hip flexion (+)/extension (−) (°)	0%	15.4 ± 6.3	23.6 ± 5.5	0.008*	<0.001*^abc^	0.582
	10%	41.3 ± 8.3	47.8 ± 5.4			
	20%	43.8 ± 8.0	50.9 ± 3.1			
Hip abduction(+)/adduction(−) (°)	0%	−2.4 ± 3.2	−3.8 ± 4.2	0.397	0.002*^ab^	0.792
	10%	−4.1 ± 6.2	−6.0 ± 5.0			
	20%	−4.7 ± 6.6	−6.7 ± 4.6			
Knee flexion (+)/extension (−) (°)	0%	32.4 ± 9.1	37.0 ± 6.1	0.093	<0.001*^abc^	0.847
	10%	72.0 ± 11.9	77.6 ± 5.2			
	20%	74.8 ± 10.4	81.0 ± 3.8			
Trunk flexion (+)/extension (−) (°)	0%	−3.1 ± 3.1	−3.9 ± 3.7	0.203	<0.001*^ab^	0.131
	10%	−0.3 ± 3.2	−2.7 ± 4.2			
	20%	0.1 ± 3.8	−2.5 ± 4.4			
Trunk lateral flexion (+)/medio lateral flexion (−) (°)	0%	3.0 ± 1.7	2.2 ± 0.8	0.033*	0.015*^b^	0.080
	10%	3.5 ± 2.0	2.0 ± 0.9			
	20%	4.5 ± 2.9	2.3 ± 1.4			

Note: *: *P* < 0.05; ^a^: Significant difference when obstacle height increased from 0% to 10%; ^b^: Significant difference when obstacle height increased from 0% to 20%; ^c^: Significant difference when obstacle height increased from 10% to 20%.

Abbreviation: FAI, Functional Ankle Instability.

Table 5 presents the results for MoS. The main effects of obstacle height were observed in the preparation phase for ML_MoS (*F* = 44.158, *P* < 0.001, *η* ^*2*^ = 0.678) and AP_MoS (*F* = 109.868, *P* < 0.001, *η* ^*2*^ = 0.840); in the Execution Phase - Toe Clearance Over Obstacle for ML_MoS (*F* = 28.783, *P* < 0.001, *η* ^*2*^ = 0.578) and AP_MoS (*F* = 166.504, *P* < 0.001, *η* ^*2*^ = 0.888); in the Execution Phase - Heel Clearance Over Obstacle for ML_MoS (*F* = 34.515, *P* < 0.001, *η* ^*2*^ = 0.622) and AP_MoS (*F* = 175.546, *P* < 0.001, *η* ^*2*^ = 0.893); and in the completion phase for ML_MoS (*F* = 9.602, *P* < 0.001, *η* ^*2*^ = 0.314) and AP_MoS (*F* = 141.731, *P* < 0.001, *η* ^*2*^ = 0.871). Post-hoc comparisons revealed that as obstacle height increased from 0% to 10% and 20%, ML_MoS significantly increased by 0.013 m (*P* < 0.001) and 0.022 m (*P* < 0.001) in the preparation phase, while AP_MoS increased by 0.073 m (*P* < 0.001) and 0.084 m (*P* < 0.001). During the Execution Phase - Heel Clearance Over Obstacle, ML_MoS increased by 0.017 m (*P* = 0.002) and 0.031 m (*P* < 0.001), and AP_MoS increased by 0.120 m (*P* < 0.001) and 0.144 m (*P* < 0.001). In the Execution Phase - Heel Clearance Over Obstacle, ML_MoS increased by 0.022 m (*P* < 0.001) and 0.038 m (*P* < 0.001), and AP_MoS increased by 0.135 m (*P* < 0.001) and 0.162 m (*P* < 0.001). In the completion phase, ML_MoS increased by 0.011 m (*P* = 0.014) and 0.013 m (*P* = 0.004), and AP_MoS increased by 0.084 m (*P* < 0.001) and 0.097 m (*P* < 0.001).

**TABLE 5:** Comparison of stability margin between the FAI group and the control group during walking over obstacles of different heights (mean ± SD).

Variables	Height	Group		*P*-value		
		FAI	Control	Group	Height	Group × height
Preparation phase						
ML_MoS (m)	0%	−0.375 ± 0.016	−0.386 ± 0.019	0.075	<0.001*^abc^	0.636
	10%	−0.360 ± 0.018	−0.375 ± 0.019			
	20%	−0.351 ± 0.019	−0.365 ± 0.017			
AP_MoS (m)	0%	−0.053 ± 0.043	−0.070 ± 0.037	0.688	<0.001*^ab^	0.255
	10%	0.010 ± 0.043	0.014 ± 0.024			
	20%	0.024 ± 0.033	0.021 ± 0.022			
Execution phase - toe clearance over obstacle						
ML_MoS (m)	0%	−0.406 ± 0.029	−0.417 ± 0.024	0.136	<0.001*^abc^	0.616
	10%	−0.385 ± 0.026	−0.403 ± 0.028			
	20%	−0.373 ± 0.028	−0.389 ± 0.022			
AP_MoS (m)	0%	−0.240 ± 0.061	−0.273 ± 0.053	0.470	<0.001*^abc^	0.241
	10%	−0.133 ± 0.071	−0.139 ± 0.051			
	20%	−0.106 ± 0.060	−0.118 ± 0.053			
Execution phase - heel clearance over obstacle						
ML_MoS (m)	0%	−0.422 ± 0.034	−0.432 ± 0.028	0.164	<0.001*^abc^	0.481
	10%	−0.394 ± 0.028	−0.415 ± 0.033			
	20%	−0.379 ± 0.030	−0.397 ± 0.024			
AP_MoS (m)	0%	−0.298 ± 0.067	−0.330 ± 0.052	0.498	<0.001*^abc^	0.374
	10%	−0.176 ± 0.076	−0.183 ± 0.058			
	20%	−0.146 ± 0.064	−0.158 ± 0.059			
Completion phase						
ML_MoS (m)	0%	0.045 ± 0.016	0.049 ± 0.019	0.046*	<0.001*^ab^	0.002*
	10%	0.048 ± 0.019	0.068 ± 0.029			
	20%	0.046 ± 0.016	0.073 ± 0.025			
AP_MoS (m)	0%	0.047 ± 0.047	0.024 ± 0.043	0.545	<0.001*^ab^	0.001*
	10%	0.105 ± 0.053	0.135 ± 0.041			
	20%	0.119 ± 0.048	0.146 ± 0.045			

Note: *: *P* < 0.05; ^a^: Significant difference when obstacle height increased from 0% to 10%; ^b^: Significant difference when obstacle height increased from 0% to 20%; ^c^: Significant difference when obstacle height increased from 10% to 20%.

Abbreviations: FAI, Functional Ankle Instability; MoS, margin of stability; ML, Medio-lateral; AP, Anterior-posterior.

Furthermore, as obstacle height increased from 10% to 20%, ML_MoS in the preparation phase significantly increased by 0.009 m (*P* < 0.001); during the execution phase (toe clearance), ML_MoS increased by 0.013 m (*P* < 0.001) and AP_MoS increased by 0.024 m (*P* = 0.010); and during the execution phase (heel clearance), ML_MoS increased by 0.016 m (*P* < 0.001) and AP_MoS increased by 0.028 m (*P* = 0.005). Additionally, a significant group difference was observed in ML_MoS during the completion phase (*F* = 4.497, *P* = 0.046, *η* ^*2*^ = 0.176), with *post hoc* analysis showing the FAI group had a 0.017 m lower MoS than the control group (*P* = 0.046).

**FIGURE 5:**
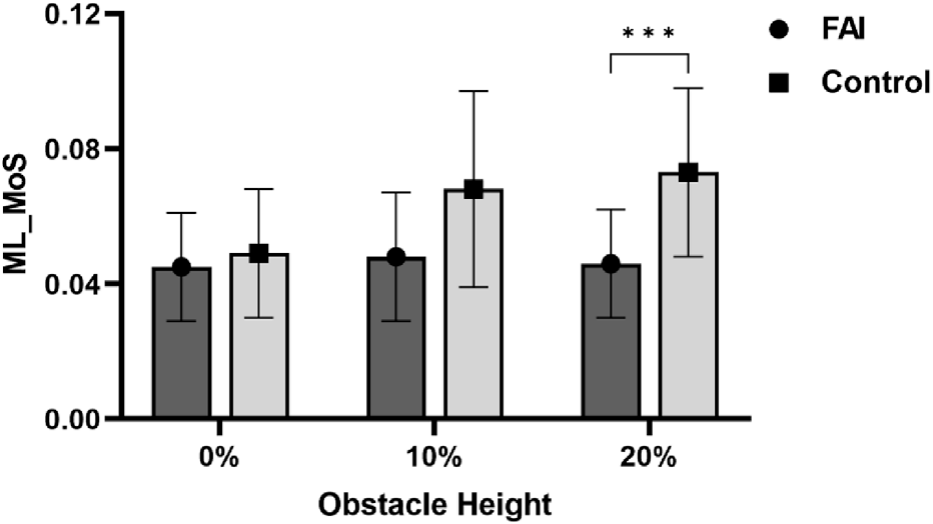
Simple analysis results in Medio-lateral Margin of Stability (ML_MoS). * *P* < 0.05.

Interaction effects between obstacle height and group were found for ML_MoS (*F* = 7.073, *P* = 0.002, *η* ^*2*^ = 0.252) and AP_MoS (*F* = 141.731, *P* < 0.001, *η* ^*2*^ = 0.871) in the completion phase. Simple effects analysis revealed that at the 20% obstacle height, the FAI group exhibited significantly lower ML_MoS than the control group (0.027 ± 0.09 m, *P* = 0.005) (Figure 5), indicating reduced ML_MoS in individuals with FAI when negotiating higher obstacles.

## 4 Discussion

### 4.1 The differences of postural control strategies in obstacle-crossing strategies between FAI and healthy individuals

#### 4.1.1 Postural control challenges during obstacle-crossing in individuals with FAI

This study examined the postural control strategies of individuals with FAI during obstacle-crossing tasks at various heights. Consistent with our Hypothesis 1, the biomechanical characteristics observed during obstacle-crossing were significantly different between individuals with FAI and healthy controls. The kinetic chain theory posits that human joints do not function in isolation but rather operate through a “proximal-distal” linkage to maintain overall mechanical efficiency and stability. When distal segments (e.g., the ankle joint) exhibit functional decline, the kinetic chain triggers compensatory synergies in proximal joints (knee, hip, pelvis, trunk) to redistribute moments, adjust center-of-mass trajectories, and ensure task completion (Cole et al., 1995; Kang and Kim, 2020). Previous studies have demonstrated that FAI induces adaptive alterations in the lower limb kinetic chain, which extends beyond the ankle to affect the knee and hip joints (Moisan et al., 2021; Son et al., 2019; Lee et al., 2022). For instance, Son et al. revealed that during level walking, individuals with FAI adopt a hip-dominant gait strategy by restricting ankle propulsive force while increasing hip power and hip flexion angles. These individuals exhibited sagittal-plane compensatory movements, marked by a 2.0° increase in hip flexion and a 22% elevation in hip extension moment (Son et al., 2019). Similarly, Kim et al. found that individuals with FAI employ analogous hip-dominant strategies during landing and jumping tasks, characterized by heightened hip extension moments, stiffness, and eccentric/concentric power (Kim et al., 2018). However, in contrast to the hip-dominant gait strategy observed by Son et al. during level walking in individuals with FAI, the results of the present study indicate that during obstacle-crossing, individuals with FAI exhibited reduced hip flexion, while the knee and ankle joints showed no significant differences compared to healthy controls. This discrepancy in findings may suggest that individuals with FAI adopt distinct postural control strategies when negotiating obstacles.

The act of crossing an obstacle, particularly as its height increases, is fundamentally a demanding proprioceptive-motor task. The requirement for precise ankle positioning, controlled weight transfer, and coordinated muscle force generation escalates with obstacle height (Yamagata et al., 2023). Concurrently, attention must be allocated to maintain continuous environmental monitoring and precise motor control (Friesen et al., 2022). Consequently, obstacle-crossing performance is critically dependent on proprioceptive integrity, with individuals exhibiting severe proprioceptive deficits often demonstrating impaired motor control during obstacle negotiation (Lu et al., 2022; Lee and Lee, 2023). In individuals with FAI, proprioceptive decline manifests as a key pathological feature. During ankle sprains, the lateral ligaments–the most frequently injured structures–sustain damage to their embedded proprioceptors, resulting in diminished or distorted proprioceptive feedback to the central nervous system (Kawabata et al., 2024). This proprioceptive information disruption directly compromises neuromuscular control efficiency, leading to reduced balance capacity (Sagnard et al., 2025), impaired joint position sense (Marinho et al., 2017), and increased postural control challenges during obstacle-crossing. Simultaneously, obstacle-crossing requires anterior flexor contraction to drive hip flexion. However, in individuals with FAI, compromised proprioception and pre-existing compensatory impairments in periarticular hip musculature (Son et al., 2019) create a mismatch: proximal muscle strength and neural drive fail to proportionally enhance to counteract distal control deficiencies. This renders the hip-dominant anti-perturbation strategy ineffective, manifesting as incomplete activation and progressive weakening of periarticular hip muscles. Consequently, an “insufficient compensation phenomenon” emerges: although hip flexion shows marginal increases with obstacle height, it remains markedly lower than in controls. Although the kinetic chain attempts compensatory adaptations, proprioceptive-motor task competition induced by FAI prevent full proximal compensation for distal deficits, ultimately sustaining stability impairments.

#### 4.1.2 Trunk compensatory mechanisms in restricted hip mobility

Notably, despite limited hip joint mobility in individuals with FAI, their vertical clearance between toes/heels and obstacles showed no significant reduction. This phenomenon may be achieved through compensatory mechanisms. The study revealed that compared to healthy controls, individuals with FAI exhibited increased trunk lateral flexion, adjusting their center of mass trajectory through lateral trunk tilting to indirectly reduce dependence on hip joint mobility. This compensation might be associated with impaired ankle proprioception resulting from previous ankle sprains in individuals with FAI. Studies have shown that individuals with compromised proprioception and diminished balance capacity tend to actively increase trunk lateral flexion and medial-lateral center of mass displacement during obstacle-crossing as compensatory adaptations to avoid collisions (Shin et al., 2015; Chou et al., 2003).

However, this compensatory strategy may initiate a dual vicious cycle: On one hand, proximal compensatory inhibition prevents adequate activation of hip muscle strength, leading to weakening of periarticular hip musculature. On the other hand, to compensate for restricted hip mobility, individuals with FAI increase trunk lateral flexion angles to modify center of mass trajectory, thereby reducing medial-lateral stability margins. This compensatory approach further hinders proper hip flexion and ultimately increases postural control difficulty when confronting unexpected external perturbations. Additionally, compensatory trunk tilting in individuals with FAI elevates energy expenditure (Takacs et al., 2014) and exacerbates dynamic instability through altered center of mass trajectory (Shin et al., 2015; Chou et al., 2003). Consequently, while this compensatory strategy preserves basic obstacle-crossing functionality, it increases movement economy costs (e.g., energy consumption) and dynamic stability risks.

### 4.2 Unique impact of increased obstacle height on individuals with FAI

#### 4.2.1 Changes in postural control due to increased obstacle height

The results demonstrate that as obstacle height increases, significant alterations occur in step length, swing velocity, phase duration distribution, joint kinematics, and MoS, indicating that obstacle height profoundly influences gait patterns, spatiotemporal parameters, joint mechanics, and stability control. These findings align with prior studies by Austin et al., who reported that higher obstacles necessitate greater postural adjustments (Austin et al., 1999), including modifications to gait (Park et al., 2012), spatiotemporal parameters (Simieli et al., 2018), joint angles (Wang et al., 2025), and stability strategies (Wang et al., 2007), to ensure successful obstacle negotiation and balance maintenance.

#### 4.2.2 Specific challenges posed by increased obstacle height for FAI individuals

Furthermore, we observed in this study that with increasing obstacle height, distinct differences in ML_MoS were observed between the FAI group and controls at the moment of obstacle-crossing completion. This result supports our Hypothesis 2, indicating that increased obstacle height leads to alterations in postural control strategies and that individuals with FAI experience greater challenges in maintaining postural stability compared to healthy controls as obstacle height increases. Notably, the FAI group exhibited significantly lower ML_MoS at 20% LL compared to controls. At lower heights (0% and 10% LL), minimal between-group differences in stability margins were observed, likely due to reduced postural challenges at these levels, which diminished the manifestation of compensatory strategies in individuals with FAI. However, at 20% LL, particularly during completion phase, ankle joint loads increased abruptly. This heightened load directly affected ankle stability and overall biomechanical characteristics, with more pronounced effects in individuals with FAI (Watanabe et al., 2021; Jang et al., 2024; Liu et al., 2025). Furthermore, impaired proprioception in individuals with FAI (Marinho et al., 2017) compromised their ability to perceive and adjust to foot loading during landing, exacerbating ankle instability (Kang et al., 2022). Given the critical role of ankle stability in maintaining dynamic stability (Simpson et al., 2019), these findings collectively suggest that FAI significantly reduces overall stability during obstacle landing phases. This height-dependent instability highlights the unique challenges faced by individuals with FAI in high-obstacle environments. While healthy controls adapt effectively to heightened demands, individuals with FAI struggle to maintain stability under increased mechanical and sensorimotor stress.

## 5 Clinical recommendations

By systematically exploring the dose-response relationship between obstacle height and obstacle height and stability deficits in FAI, which provides valuable insights into the challenging stability issues faced by individuals with FAI during obstacle-crossing. In contrast, healthy controls demonstrated superior adaptability to these environments.

Current rehabilitation for individuals with FAI primarily utilizes balance and proprioceptive training. While these approaches demonstrably improve dynamic stability and subjective outcomes (Yekdaneh and Mutlu, 2024), their benefits remain limited, and no single intervention has emerged as clearly optimal (Tedeschi et al., 2024). Moreover, the adaptive responses of the central nervous system to ankle injury in individuals with FAI alter postural control strategies, thereby increasing their risk of subsequent injury during complex movements (Wang et al., 2024a). This study found that FAI involves deficits in proprioception and balance control, significantly increasing the difficulty of maintaining postural stability during obstacle-crossing. Consequently, obstacle-crossing training offers a valuable functional adjunct to rehabilitation. As a targeted functional activity, this training simulates real-world environmental demands, effectively enhancing an individual’s obstacle avoidance capability (Weerdesteyn et al., 2008), spatial awareness (Pramodhyakul et al., 2013; Barral, 2023), and overall postural control (Pramodhyakul et al., 2013). Importantly, the objective performance feedback inherent in obstacle-crossing tasks (e.g., success/failure, clearance height) may enhance individuals with FAI’s motivation and rehabilitation adherence by providing tangible evidence of functional improvement. Incorporating obstacle-crossing training into rehabilitation protocols, integrated with sensorimotor and motor control strategies, is essential in fostering neural remodeling associated with ankle ligament injuries (Maricot et al., 2023), thus also mitigating fall risks during complex daily activities for individuals with FAI.

## 6 Limitations

This study still has some limitations: (1) To avoid potential confounding effects of sex and age differences, the current cohort was restricted to young male participants only. Future research should include female participants and more diverse age groups to improve generalizability and reliability; (2) The experimental design required participants to perform the task with the affected limb crossing the obstacle and the unaffected limb supporting the body. Differences in strategy when reversing this pattern (unaffected limb crossing, affected limb supporting) remain unexplored; (3) The study only tested obstacle heights of 0%, 10%, and 20% of LL. The effects of higher obstacles on individuals with FAI remain unclear.

## 7 Conclusion

Individuals with FAI employed unique compensatory mechanisms during obstacle-crossing with the affected limb. Compared to healthy controls, their hip joints could not be sufficiently activated, thus adopting a trunk compensatory strategy, characterized by significantly reduced hip flexion angles and compensatory increases in trunk lateral flexion. As obstacle height increased, individuals with FAI demonstrated decreased stability during landing phases, particularly showing a significant reduction in lateral stability at higher obstacle heights. These findings emphasize the impact of FAI on balance control and motor strategy adaptation during obstacle negotiation, providing insights into mitigating reinjury risks in this population.

## Data Availability

The raw data supporting the conclusions of this article will be made available by the authors, without undue reservation.
